# Supplementary material for: Akt Signaling Pathway Is Activated in the Minor Salivary Glands of Patients with Primary Sjögren’s Syndrome
Source: Int J Mol Sci. 2021 Dec 14;22(24):13441. doi: 10.3390/ijms222413441 (PMC8709495; doi:10.3390/ijms222413441)
Supplement: Supplementary file 1 [file ijms-22-13441-s001.zip › ijms-1498647-supplementary.pdf]

Supplementary Figure

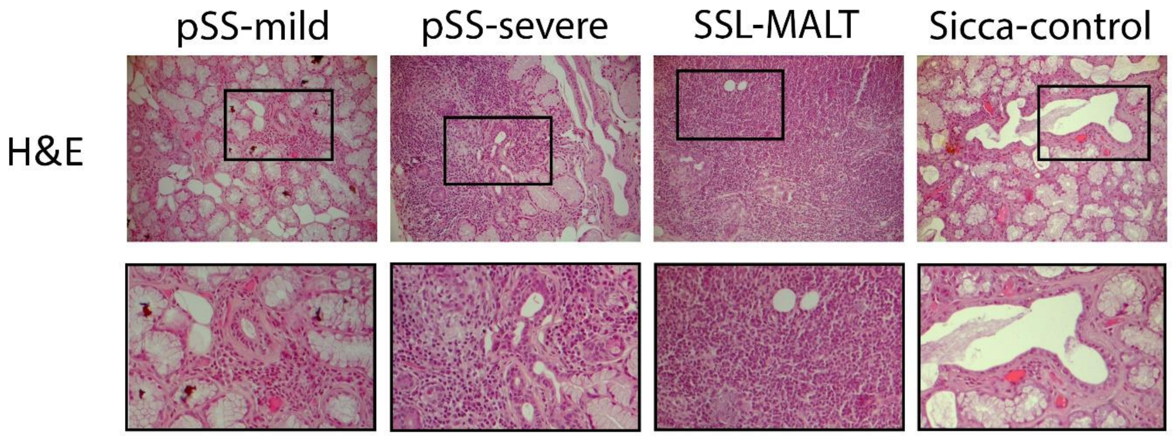

Suppl. Figure S1. Representative pictures of Hematoxylin and Eosin staining of the MSGs of pSS patients with mild or severe MSG inflammatory infiltrates (pSS-mild and pSS-severe, respectively) or with NHL (SSL), as well as a sicca-complaining control *(sicca-control)*. The lower panel represent detail of pictures presented in the upper panel (square). The pictures correspond to those presented in Figure 1. Original objective magnification: x20.
